# BNT162b2 coronavirus disease-2019 vaccination accelerated rheumatoid arthritis disease activity in chronic eosinophilic pneumonia: A case report

**DOI:** 10.1097/MD.0000000000030806

**Published:** 2022-09-30

**Authors:** Moeko Murano Morikawa, Masanori Harada, Ei Kishimoto, Kosuke Suzuki, Emiko Nakagawa, Toshiya Hiramatsu, Shogo Nakai, Yurina Murakami, Koji Nishimoto, Sayomi Matsushima, Tomohiro Uto, Shiro Imokawa

**Affiliations:** a Department of Respiratory Medicine, Iwata City Hospital, Iwata, Shizuoka Province, Japan.

**Keywords:** chronic eosinophilic pneumonia, COVID-19 vaccination, M2-phenotype cytokines, rheumatoid arthritis

## Abstract

The relationship between rheumatoid arthritis (RA) and eosinophilic inflammation is unclear. According to recent studies, it has been suggested that T helper 2 cell responses play a role in the inhibition of RA. It is unclear how the immunological response after coronavirus disease-2019 (COVID-19) vaccination affects T cell immune reactions.

**Patient concerns and diagnoses:**

Here, we report the case of an 88-year-old woman diagnosed with RA and chronic eosinophilic pneumonia (CEP). She was diagnosed with CEP about 20 years ago, and, through steroid treatment, she improved and had no relapse for 16 years. At the time of diagnosis of CEP, the rheumatoid factor (RF) was increased; however, there were no joint symptoms. After receiving the COVID-19 vaccine, joint and respiratory symptoms gradually worsened. Laboratory examinations showed increased RF, anti-cyclin citrullinated peptide antibody, and peripheral absolute eosinophil count. Musculoskeletal ultrasonography showed synovitis.

**Intervention and outcome:**

Methylprednisolone pulse therapy improved respiratory and joint symptoms immediately; RA and CEP stabilized with no relapses.

**Lessons:**

Eosinophilic and rheumatoid reactions following COVID-19 vaccination were an-reported adverse events. Eosinophilic inflammation might be reflected on an anti-inflammatory reaction in initial phase of RA.

## 1. Introduction

Rheumatoid arthritis (RA) is a severe inflammatory autoimmune disease that targets the synovial membrane, cartilage, and bone. The main pathological prevailing dogma is a pro-inflammatory M1-phenotype, leading to the production of pro-inflammatory mediators such as tumor necrosis factor, interleukin (IL)-1 and IL-6,^[1]^ accompanied by a reduction in regulatory and anti-inflammatory M2-phenotype cytokines, such as transforming growth factor-β (TGFβ), IL-4, IL-13, and IL-10.^[2]^ Recent studies have shown that T helper 2 (TH2) cells activated by IL-4, IL-5, and IL-13 play a role in the inhibition of RA, substantially down-regulating pro-inflammatory cytokines.^[3]^ However, how TH2 cell responses inhibit arthritis remains poorly understood.

Today, the coronavirus disease-2019 (COVID-19) pandemic has worsened, causing high mortality.^[4]^ The early immune response, which consists of neutralizing antibodies against SARS-CoV-2, has a primarily protective role; therefore, the prophylactic vaccination is thought to be one of the central statistical dogmas against the COVID-19 pandemic worldwide.^[5]^ The BNT162b2 mRNA vaccine encodes the production of the SARS-CoV-2 spike protein, which is the primary target for neutralizing antibodies, which are also generated from natural infection.^[6]^ The vaccine activates spike-protein-specific T cells and adaptive immunity against SARS-CoV-2. Transient vaccine-related reactogenicity events such as shoulder injury, axillary lymphadenopathy, ventricular arryhthmia, and leg paresthesia have been reported; however, other types of immunological adverse events are poorly understood.

Here, we present the first case of RA disease activity with eosinophil infiltration accelerated after BNT162b2 mRNA coronavirus disease-2019 (COVID-19) vaccination.

## 2. Case report

**Figure 1.**
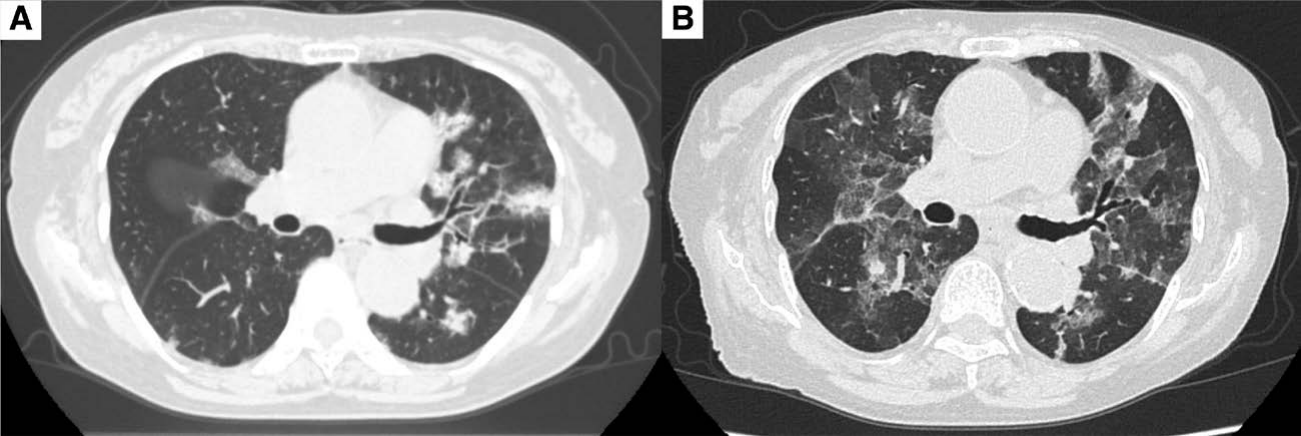
(A) Chest computed tomography image taken approximately 20 years ago, showing multiple patchy areas in both lungs. (B) After vaccination, image findings were similar to those 20 years ago.

An 88-year-old Japanese woman presented with bilateral joint pain and morning stiffness of the metacarpophalangeal joints 2 months prior to her admission to our hospital. She was diagnosed with chronic eosinophilic pneumonia (CEP) 20 years ago. At the time of diagnosis, chest computed tomography (CT) examination showed randomly distributed patchy consolidations in the left and right lungs, as shown in Figure 1A. Her peripheral absolute eosinophil count (pAEC) and serum rheumatoid facture (RF) were elevated at 4760/μL and 232 IU/mL, respectively. However, the patient had no relevant joint symptoms. The bronchoalveolar lavage (BAL) examination showed a high eosinophil count (72%) and eosinophil tissue infiltration. Based on these results, she was diagnosed with CEP. Upon initiating oral prednisolone therapy (40 mg/day), her respiratory symptoms and CT findings gradually improved. No relapse was observed, despite the patient not being under medication.

**Figure 2.**
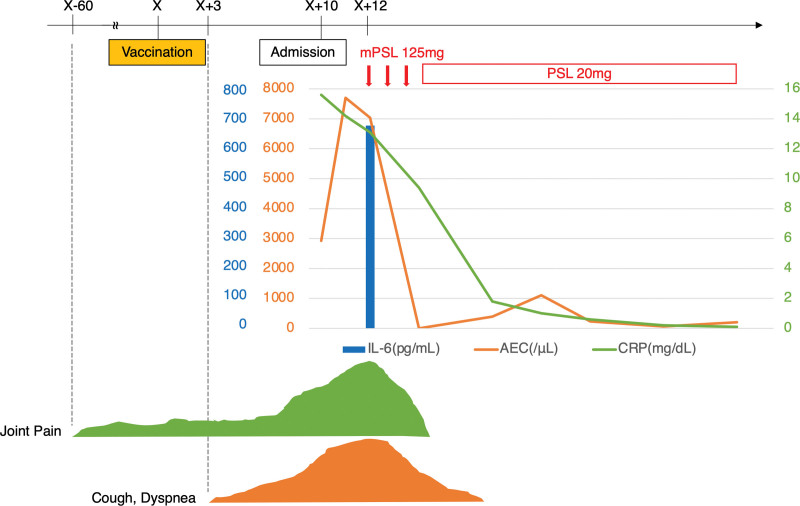
Clinical course from vaccination. Day X indicates the day of vaccination. AEC = absolute eosinophil count, IL-6 = interleukin-6, mPSL = methylprednisolone, PSL = prednisolone.

**Figure 3.**
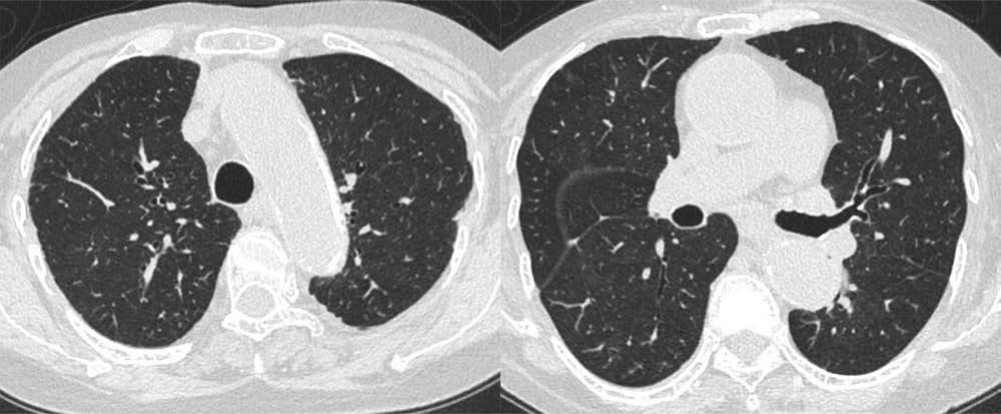
A chest computed tomography image after the steroid treatment.

**Figure 4.**
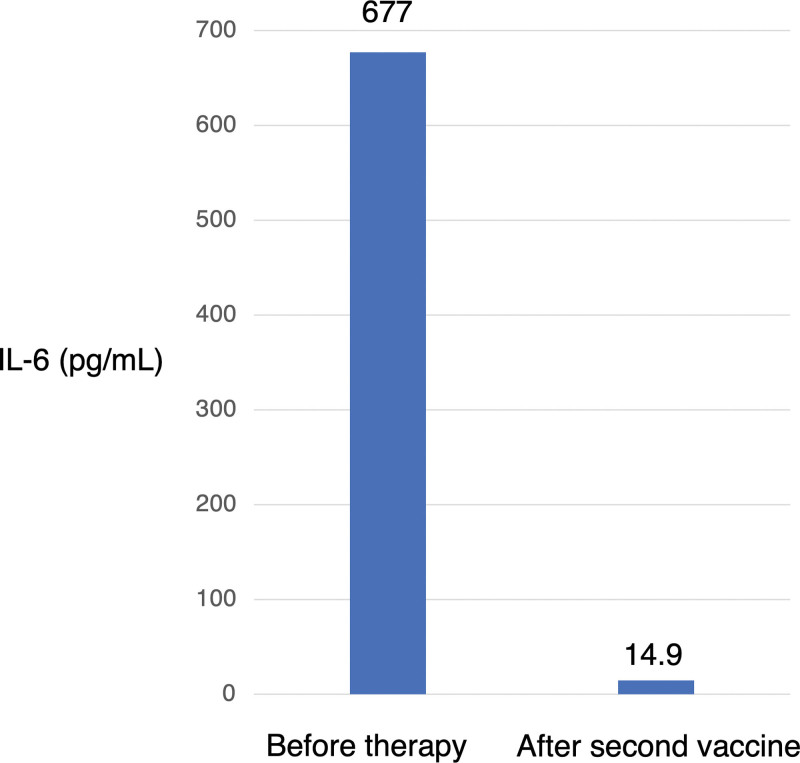
Total serum IL-6. Left side shows results before steroid therapy, while the right side shows results after the second dose of the vaccine. IL-6 = interleukin-6.

Ten days prior to her admission to our hospital, she received the BNT162b2 mRNA COVID-19 vaccine. Three days later, she developed fever, dyspnea, productive cough, and general malaise; these symptoms gradually worsened, and she was admitted to our hospital. Chest CT showed multiple ground-glass opacities and consolidations in the both lung fields (Fig. 1B). These findings were similar to those of the initially diagnosed CEP. Laboratory examinations showed an elevated RF (107 IU/mL), anti-cyclin citrullinated peptide antibody (61.8 U/mL), C-reactive protein (15.6 mg/dL), and serum IL-6 (677 pg/mL). The pAEC was also increased (2955/µL). Furthermore, musculoskeletal ultrasonography of the wrist region was conducted to confirm the diagnosis of RA. Grayscale ultrasonography showed grade 3 synovitis, which included both synovial hypertrophy and effusion. Power Doppler ultrasonography also showed grade 3 active synovitis (Figure S1, Supplemental Digital Content, http://links.lww.com/MD/H395). These results satisfied the 2010 American College of Rheumatology/European League Against Rheumatism classification criteria for rheumatoid arthritis,^[7]^ and she was diagnosed with rheumatoid arthritis. Due to her worsening respiratory condition, oxygen therapy was needed. Therefore, methylprednisolone pulse therapy was immediately initiated. After her respiratory condition had stabilized, bronchoscopy was performed because CEP was a differential diagnosis due to the abnormal CT findings. The eosinophil cell counts in the BAL fluid were elevated (9.2%) even during steroid therapy. However, there was no bacterial evidence, leading to a CEP suspicion. The prednisolone treatment was continued. After presnisolone discontinuation, her imaging findings, respiratory symptoms, and joint pain improved (Figs. 2 and 3). She was able to receive the second dose of the vaccine. Her general condition and pAEC remained normal, and her serum IL-6 level decreased (14.9 pg/mL) (Fig. 4).

## 3. Discussion

This was the first documented case of RA and eosinophil infiltration with CEP after BNT162b2 mRNA COVID-19 vaccination. This case depicted several similarities between the immune profile of RA and COVID-19. IL-6 or Janus kinase (JAK) inhibitors were reportedly efficacious against COVID-19 in the clinical setting, and they have also been used in RA therapy.^[8]^ In the late phase of COVID-19, the cytokine storm is the main cause of lung injury, and several immune factors, such as IL-6, JAK1/2, and GM-CSF, were reportedly common signaling cascades, like those in RA. These mediators have also been reported in COVID-19 patients from Wuhan, China.^[9]^ In our case, the immune response was caused by SARS-CoV-2 vaccination rather than infection. Previous clinical studies have not reported on cytokine release syndrome (CRS).^[6]^ However, the cytokine reactions against SARS-CoV-2 spike proteins were likely similar to those against COVID-19 infection.

Recently, a case of CRS following BNT162b2 vaccination was reported in a colorectal cancer patient treated with anti-PD-1 monotherapy.^[10]^ In this case, a cytokine storm was observed only 5 days after vaccination. This patient received a PD-1 inhibitor for 22 months, and then BNT162b2 vaccination was administered. The initial cytokine profile indicated both type 1 helper T cells and macrophage activation. This vaccine-induced immune response was likely induced under activated T cell immune reactive conditions by a PD-1 inhibitor; in our case, the allergic condition of CEP easily induced similar immune reactions.

The correlation between RA and eosinophil infiltration has not been fully understood. A recent study reported that hypereosinophilic syndrome, a rare adverse event of anti-cytokine treatment in RA, was resolved after JAK inhibitor.^[11]^ Similarly, GM-CSF was reportedly involved in the pathogenesis of RA, and^[8]^ serum eosinophils by GM-CSF have also been associated with phosphorylation of JAK 2.^[12]^ These results suggest that induced GM-CSF expression was a common pathological factor between RA and eosinophilic diseases. Therefore, the BNT162b2 vaccination emphasized this common pathological signaling cascade.

Osteoclasts are multinuclear bone-resorbing cells differentiated from monocyte/macrophage lineage cells and depending on the receptor activator of nuclear factor κB (RANKL).^[13]^ Osteoclasts differentiation plays a key role in a steady-state RA and is controlled by Group 2 innate lymphoid cells in the bone marrow (BM ILC2s). Naïve BM ILC2s show high RANKL expression up-regulated by IL-2, IL-7, and all-trans retinoic acid, while BM ILC2s activated by IL-33 suppress RANKL expression and induce the differentiation of progenitors; BM-derived monocyte/macrophage lineage cells (BMMs), into M2 macrophage via abundant IL-13 and GM-CSF production.^[14]^ Andreev et al reported that the ILC2s- IL-5 axis stimulated by IL-33 and IL-25 induced the production of a specific subtype of eosinophils, regulatory eosinophils (rEOS), in the joint that stimulated arthritis resolution. This synovial rEOS expanded on systemic up-regulation of IL-5 released by lung ILC2s in patients with RA remission, and anti-IL-5 antibody therapy induced the relapse of arthritis. So rEOS induced by ILC2s could play a relevant role in RA remission in the active phase of the disease.^[15]^

According to previous reports, ILC2s seem to play a “double-edged sword” role in RA disease activity. In the early steady-state phase of RA, BM ILC2s contribute to BMMs differentiation to osteoclasts; however, in the active phase of RA, BM ILC2s suppress RANKL expression, BMMs differentiation into not osteoclasts, but M2 macrophage. Subsequently, ILC2s induced by IL-33 produce rEOS via IL-5 over-expression, leading to RA remission. Therefore, in our case, BNT162b2 vaccination might have induced RA-related cytokine production and triggered RA disease initiation. We hypothesize that BM ILC2s are an essential factor for RA disease initiation, and subsequent IL-33-induced ILC2s production is regulated through a negative feedback loop, leading to the peripheral eosinophilia, including rEOS accumulation in joint.

Vaccination is one of the useful and essential therapies to control the global COVID-19 pandemic; however, it is crucial to understand its adverse events. Eosinophilic and rheumatoid reactions following BNT162b2 vaccination were newly reported adverse events. Although the causality of the correlation between vaccination and the development of these reactions is not established, this reaction occurred transiently and was completely recovered (Figs. 2 and 3). Both innate immune and Th cell adaptive reactions to COVID-19 might be a trigger of RA, subsequently inducing activation of disease activity of CEP. Initial disease activity of rheumatoid arthritis might induce the M2-phenotype reaction. A prospective clinical study is needed to clarify the development of immunological reactions after COVID-19 vaccination. Furthermore, the correlation between RA and eosinophils should be considered in the clinical setting.

## Acknowledgment

We would like to thank Editage (www.editage.com) for English language editing.

## Author contributions

MM and MH managed the patient, created the figures, and collected the laboratory data. MM and MH wrote the manuscript. MM, MH, EK, KS, EN, TH, SN, YM, KN, SM, TU, and SI contributed to the discussion of the results and reviewed the manuscript. All authors read and approved the final manuscript.

**Conceptualization:** Moeko Murano Morikawa, Masanori Harada, Shogo Nakai, Koji Nishimoto, Sayomi Matsushima, Tomohiro Uto, Shiro Imokawa.

**Data curation:** Moeko Murano Morikawa, Masanori Harada, Ei Kishimoto, Kosuke Suzuki, Emiko Nakagawa, Toshiya Hiramatsu, Shogo Nakai, Yurina Murakami, Koji Nishimoto, Sayomi Matsushima, Shiro Imokawa.

**Formal analysis:** Masanori Harada.

**Investigation:** Moeko Murano Morikawa, Masanori Harada, Koji Nishimoto.

**Project administration:** Masanori Harada, Shiro Imokawa.

**Supervision:** Masanori Harada, Tomohiro Uto, Shiro Imokawa.

**Visualization:** Koji Nishimoto.

**Writing – original draft:** Moeko Murano Morikawa, Masanori Harada, Ei Kishimoto, Kosuke Suzuki, Emiko Nakagawa, Toshiya Hiramatsu, Shogo Nakai, Yurina Murakami, Koji Nishimoto, Sayomi Matsushima, Tomohiro Uto, Shiro Imokawa.

**Writing – review & editing:** Moeko Murano Morikawa, Masanori Harada, Ei Kishimoto, Kosuke Suzuki, Emiko Nakagawa, Toshiya Hiramatsu, Shogo Nakai, Yurina Murakami, Koji Nishimoto, Sayomi Matsushima, Tomohiro Uto, Shiro Imokawa.

## Supplementary Material


