# Supplementary material for: BNT162b2 coronavirus disease-2019 vaccination accelerated rheumatoid arthritis disease activity in chronic eosinophilic pneumonia: A case report
Source: Medicine (Baltimore). 2022 Sep 30;101(39):e30806. doi: 10.1097/MD.0000000000030806 (PMC9524534; doi:10.1097/MD.0000000000030806)

Supplementary figure:

Musculoskeletal ultrasonography of the wrist region. A: Grayscale ultrasonography showed synovial hypertrophy and effusion (asterisk). B: Power Doppler ultrasonography showed intra-articluar blood flow representing an active inflamed joint (arrow). Both indicated grade 3 synovitis.

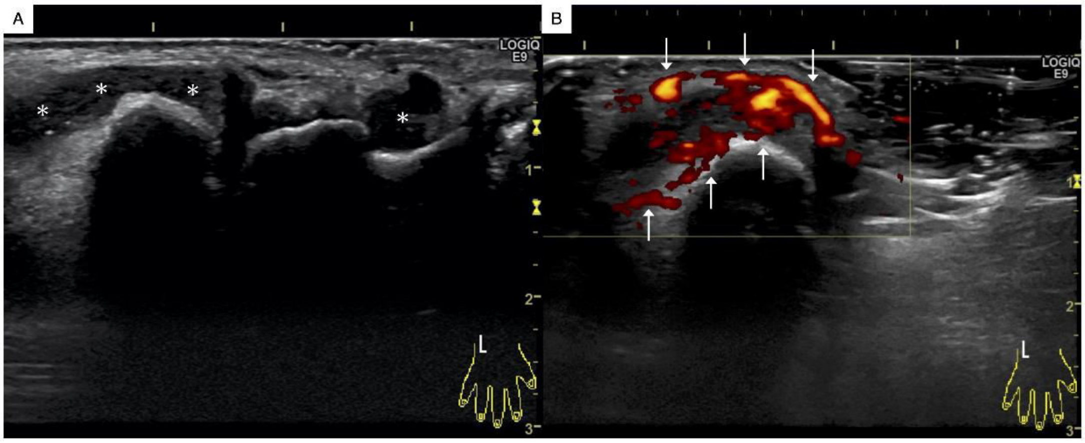

Supplement: Supplementary file 1 [file medi-101-e30806-s001.pdf]
